# Relationship Between Sleep Quality and Quality of Life: Analysis of Hepatocellular Carcinoma Survivors

**DOI:** 10.7759/cureus.63883

**Published:** 2024-07-05

**Authors:** Toru Ishikawa, Narumi Arita, Yusuke Matsuhashi, Terasu Honma

**Affiliations:** 1 Department of Gastroenterology and Hepatology, Saiseikai Niigata Hospital, Niigata, JPN; 2 Department of Nursing, Saiseikai Niigata Hospital, Niigata, JPN

**Keywords:** survivors, hepatocellular carcinoma, 36-item short form survey, quality of life, pittsburgh sleep quality index

## Abstract

Background: We analyzed the correlation between the Pittsburgh Sleep Quality Index (PSQI) subcategories (sleep quality, sleep latency, sleep duration, sleep efficiency, sleep disturbance, sleep medications, and daytime dysfunction) and a comprehensive measure of quality of life (QOL), the 36-Item Short Form Survey (SF-36) items, in patients with hepatocellular carcinoma (HCC) to determine the components that require intervention to improve QOL.

Methods: A total of 75 patients with recurrent HCC admitted to our hospital between May 2021 and May 2023 were included in this study. The QOL score was used for the SF-36 items, and the sleep disorder score was used for PSQI questionnaires.

Results: Correlations were found between sleep quality, sleep disturbance, and SF-36 for all QOL items and between sleep onset time and SF-36 for six QOL items: bodily pain, mental health, physical functioning, role-emotional, role-physical, and vitality. Correlations between daytime dysfunction and SF-36 were found for all QOL items, except for physical functioning. No correlation was found between sleep duration, sleep efficiency, sleep medications, and SF-36 for any QOL item.

Conclusion: Sleep duration, sleep efficiency, and sleep medications may not contribute to QOL improvement in patients with HCC and sleep disturbances. Factors that improve sleep quality and sleep difficulty may contribute to QOL improvement. Therapeutic interventions aimed at improving general health and social functioning for sleep latency and physical functioning for daytime arousal difficulty are required.

## Introduction

Sleep disturbances in patients with cancer vary based on the stage and type of cancer but frequently occur in 30-50% of patients, most of whom have chronic insomnia lasting > six months [1]. The rate of sleep disturbance in patients with cancer is estimated to be two to three times higher than that in patients without cancer [2,3].

In patients with hepatocellular carcinoma (HCC), the rate of intrahepatic recurrence within two years of diagnosis and treatment is as high as 28.8%, making recurrence extremely common [4]. Radiofrequency ablation (RFA) and transcatheter arterial chemoembolization (TACE), which are minimally invasive and repeatable, are often indicated for the treatment of recurrent HCC and have significantly improved survival rates. Symptoms associated with recurrence are rare; however, moderate post-treatment fatigue often persists [5].

The National Coalition for Cancer Survivorship defines a person diagnosed with cancer as a survivor from the time of diagnosis until death [6]. HCC survivors are often concerned regarding the impact of an unstable state of mind and increased fatigue on sleep when they experience relapse and undergo subsequent treatment. Studies on sleep in HCC survivors are scarce [7] and its association with quality of life (QOL) has not been reported. Understanding the actual condition of the sleep subcategories and the factors affecting QOL will provide clues to support the improvement of QOL. Therefore, this study aimed to analyze the correlation between the Pittsburgh Sleep Quality Index (PSQI) subcategories and a comprehensive measure of QOL in patients with recurrent HCC to determine the components that require intervention to improve QOL.

## Materials and methods

Recurrent HCC admitted to our hospital between May 2021 and May 2023 for the treatment of recurrent HCC and who were able to complete the 36-Item Short Form Survey (SF-36) [8] and Pittsburgh Sleep Quality Index (PSQI) questionnaires [9] in the same session were included in this study.

The inclusion criteria were as follows: (1) patients who were aware of HCC recurrence; (2) patients scheduled to undergo RFA or TACE; (3) patients who had no diseases requiring hospitalization other than HCC; (4) patients with no metal allergy; and (5) performance status of Eastern Cooperative Oncology Group score 0-2.

The exclusion criteria were as follows: 1) patients whose systemic therapy had been administered; 2) patients who had complicated other cancers; and 3) patients whose SF36 and PSQI responses could not be obtained on the same day.

The Medical Outcomes Study SF-36 was used to assess health-related QOL. The SF-36 questionnaire included four items assessing physical QOL, namely, physical functioning (PF), role-physical (RP), bodily pain (BP), and general health perception (GH), and four items assessing mental health QOL, namely, vitality (VT), social functioning (SF), role-emotional (RE), and mental health (MH). In total, eight subscales were assessed.

The PSQI is a self-administered questionnaire used universally in clinical and epidemiological studies to evaluate sleep and its quality. The PSQI evaluates the following items: sleep quality (overall subjective assessment of sleep), sleep duration (assessment of total sleep time), falling asleep time (assessment of the ease of falling asleep), sleep efficiency (assessment of actual sleep time relative to bedtime), sleep difficulty (rating the degree of mid-sleep awakening), use of sleep aids (rating the frequency of taking medicine to induce sleep), and disturbance to daily life due to daytime sleepiness and other problems (rating daytime sleepiness associated with sleep and other problems).

Statement of ethics

The study protocols were approved by the Institutional Review Board of Saiseikai Niigata Hospital and conducted in accordance with the principles of the Declaration of Helsinki (as revised in 2013). Before participating in this study, written informed consent was obtained from all patients.

Statistical analysis

Pearson’s correlation was used to analyze the correlation between PSQI and SF-36. Statistical significance was set at p<0.05. All statistical analyses were performed using EZR (Saitama Medical Centre, Jichi Medical University, Shimotsuke, Japan), a graphical user interface for R version 3.2.2 (The R Foundation for Statistical Computing, Vienna, Austria) [10].

## Results

In total, 75 patients have a mean age of 70.3 ± 8.27 years (59 male and 16 female patients). Among the participants in this study, the background liver factors were as follows: 12 cases of hepatitis C virus (HCV), 13 cases of hepatitis B virus (HBV), and 50 non-HBV/non-HCV cases. The Child-Pugh scores were as follows: (A) 55 cases; (B) 17 cases; and (C) three cases. Albumin-bilirubin grades were as follows: (1) 30 cases; (2) 38 cases; and (3) seven cases (Table 1).

**Table 1:** Demographic and clinical characteristics of 75 patients with recurrent hepatocellular carcinoma

Variables	n=75
Age (years)	70.3±8.27
Sex, n (%)	
Male	59 (78.6%)
Female	16 (21.4%)
Etiology, n(%)	
HBV	12 (16.0%)
HCV	12 (16.0%)
Non-HBV and non-HCV	51 (68.0%)
Child-Pugh n(%)	
A	55 (73.3%)
B	17 (22.7%)
C	3 (4.0%)
ALBI grade n(%)	
1	30 (40.0%)
2	38 (50.7%)
3	7 (9.3%)

In the evaluation of sleep quality, sleep difficulty, and SF-36 among the seven PSQI subcategories, correlations were found for all eight QOL items. In the evaluation of time to fall asleep and SF-36, correlations were found for six QOL items: BP, MH, PF, RE, RP, and VT, but not for GH and SF. In the evaluation of daytime arousal difficulty and SF-36, correlations were found for seven QOL items: BP, GH, MH, RE, RP, SF, and VT, but not for PF. No correlations were found between sleep duration, sleep efficiency, use of sleep aids, and SF-36 for any of the eight QOL items (Table 2).

**Table 2:** Correlation between Pittsburgh Sleep Quality Index subclasses and SF-36 in patients with hepatocellular carcinoma

	Sleep quality	Time to fall asleep	Sleep duration	Sleep efficiency	Sleep difficulty	Use of sleep aids	Daytime arousal difficulty
BP	r=-0.344 (p<0.01)	r=-0.375 (p<0.01)	r=-0.1 (p=0.39)	r=-0.11 (p=0.34)	r=-0.386 (p<0.01)	r=0.0621 (p=0.59)	r=-0.54 (p<0.01)
GH	r=-0.475 (p<0.01)	r=-0.21 (p=0.07)	r=-0.131 (p=0.26)	r=-0.0777 (p=0.50)	r=-0.372 (p<0.01)	r=-0.0432 (p=0.71)	r=-0.361 (p<0.01)
MH	r=-0.345 (p<0.01)	r=-0.23 (p=0.04)	r=-0.042 (p=0.72)	r=0.835 (p=0.47)	r=-0.366 (p<0.01)	r=-0.198 (p=0.09)	r=-0.366 (p<0.01)
PF	r=-0.179 (p=0.12)	r=-0.281 (p=0.014)	r=-0.496 (p=0.67)	r=-0.0719 (p=0.53)	r=-0.222 (p<0.01)	r=0.0231 (p=0.84)	r=-0.181 (p=0.12)
RE	r=-0.387 (p<0.01)	r=-0.399 (p<0.01)	r=-0.02 (p=0.86)	r=-0.137 (p=0.23)	r=-0.31 (p<0.01)	r=-0.0448 (p=0.69)	r=-0.508 (p<0.01)
RP	r=-0.326 (p<0.01)	r=-0.338 (p<0.01)	r=-0.0493 (p=0.67)	r=-0.173 (p=0.13)	r=-0.291 (p<0.01)	r=-0.0528 (p=0.65)	r=-0.374 (p<0.01)
SF	r=-0.255 (p=0.02)	r=-0.201 (p=0.084)	r=-0.0928 (p=0.42)	r=0.109 (p=0.35)	r=-0.367 (p<0.01)	r=-0.132 (p=0.25)	r=-0.519 (p<0.01)
VT	r=-0.375 (p<0.01)	r=-0.287 (p=0.013)	r=-0.0507 (p=0.67)	r=0.0724 (p=0.53)	r=-0.424 (p<0.01)	r=-0.132 (p=0.26)	r=-0.418 (p<0.01)

## Discussion

In recent years, sleep disturbances have been reported to occur at high rates during cancer treatment [2]. Sleep disturbances also act as a deterrent to continued treatment and increase the risk of developing delirium and mood disorders. Several epidemiological studies of the general population have shown that sleep disorders are also a major factor in reducing QOL, while some reports have shown that the presence of a sleep disorder alone is associated with reduced QOL and that the more severe the sleep disorder, the greater is the reduction in QOL [11].

The PSQI, developed in 1988, is commonly used to assess sleep disturbance [9], and its usefulness in the assessment of patients with chronic liver disease (CLD) is well-established [12-14]. However, studies on HCC, a terminal manifestation of CLD, have not been reported. Although sleep disturbances are less important than other symptoms during cancer treatment, they are highly prevalent during cancer treatment. Therefore, in this study, we investigated the clinical importance of sleep disturbances during the treatment of recurrent HCC and the subcategories of sleep disorders in terms of QOL during cancer treatment.

In this study, correlations between the PSQI subcategories and SF-36 were found for all eight QOL items, while correlations between sleep quality, sleep difficulty, and SF-36 were found for BP, MH, PF, RE, RP, and VT, but not for GH and SF. Furthermore, correlations were found between daytime arousal difficulty and SF-36 for seven QOL items: BP, GH, MH, RE, RP, SF, and VT, but not for PF. However, no correlations were found between SF-36, sleep duration, sleep efficiency, and use of aids for any of the eight QOL items.

Sleep disturbances in patients with HCC due to CLD may lead to a reduced QOL. In general, the use of sleep aids may be considered to address sleep difficulties in patients with HCC; however, the lack of correlation between the use of sleep aids and SF-36 suggests that the easy use of sleep medication is not a solution for sleep disorders in patients with HCC.

Sleep disorders are often treated with sleeping pills; however, these drugs depress the central nervous system and may induce hepatic encephalopathy in patients with HCC due to CLD [15,16]. Therefore, the treatment for sleep disturbances in patients with HCC should be tailored to the underlying cause. However, considering the risk of hepatic encephalopathy, the use of sleeping pills should be avoided, as evidenced by the lack of a correlation between QOL and sleep disturbances in the present study.

The treatment of liver-related complications, such as hepatic encephalopathy and muscle spasm, can improve sleep disturbances in patients with CLD [17,18]; however, further studies are necessary to determine whether the improvement of symptoms in patients with HCC, other than the use of sleep aids, is effective in improving sleep disturbances and QOL in these patients.

This study has some limitations. First, this was a retrospective study with a small number of patients. Second, the patients on systemic therapy for HCC were not included. Third, the number of recurrences has not been analyzed in this study.

In the future, sleep disorders and QOL should be analyzed in various HCC treatments in a large number of cases.

## Conclusions

In order to improve the QOL of HCC survivors, it is important to understand their sleep status based on the PSQI and provide therapeutic intervention according to their subclass. Based on the analysis of PSQI and QOL, it is not expected that sleeping pills will solve the problem of recurrence of HCC. Further analysis of the causes of HCC cases related to sleep disorders is required in the future.
